# The Concentration of Fibronectin and MMP-1 in Patients with Alzheimer’s Disease in Relation to the Selected Antioxidant Elements and Eating Habits

**DOI:** 10.3390/jcm11216360

**Published:** 2022-10-27

**Authors:** Sylwia Bogdan, Anna Puścion-Jakubik, Katarzyna Klimiuk, Katarzyna Socha, Jan Kochanowicz, Ewa Gorodkiewicz

**Affiliations:** 1 Bioanalysis Laboratory, Faculty of Chemistry, University of Bialystok, Ciołkowskiego 1K, 15-245 Bialystok, Poland; 2 Department of Bromatology, Faculty of Pharmacy with the Division of Laboratory Medicine, Medical University of Białystok, Mickiewicza 2D Street, 15-222 Bialystok, Poland; 3 Podlasie Center of Psychogeriatrics, Swobodna 38 Street, 15-756 Bialystok, Poland; 4 Department of Neurology, Medical University of Białystok, M. Skłodowskiej-Curie 24a Street, 15-276 Bialystok, Poland

**Keywords:** copper, selenium, zinc, total antioxidant status, fibronectin, MMP-1, eating habits

## Abstract

Alzheimer’s disease (AD) is a neurodegenerative disease and the most common form of dementia in the elderly. In recent years, markers of this disease have been researched, with an emphasis on prophylaxis. The aim of this study is to evaluate the concentration of fibronectin and MMP-1 in serums in relation to levels of antioxidant elements, as well as eating habits in the group of patients with AD (*n* = 110). The control group consisted of 60 healthy people. The conducted studies showed that patients with AD are characterized by a significantly higher median concentration of fibronectin compared to healthy subjects (652.06 vs. 268.31 µg/mL), but a significantly lower median of MMP-1 (4.62 vs. 18.09 ng/mL). Significant inverse correlations between MMP-1 and the concentration of antioxidant elements, as well as positive correlations between MMP-1 vs. Total Antioxidant Status (TAS) and MMSE, were observed. Multiple regressions showed that the concentration of fibronectin can be explained in 28% cases by eating habits, and by MMP-1 in 25%. Nutritional modifications to reduce the consumption of fruit, meat and processed products can be part of AD prevention.

## 1. Introduction

Alzheimer’s disease (AD) is a progressive neurodegenerative disease that is the most common form of dementia in the elderly. It is most common in people over 65, but it can also occur in younger people. Before the first symptoms appear, the disease may progress for years without diagnosis. Life expectancy after diagnosis is 7–10 years. Risk factors for AD include: older age, female gender, diabetes, and low education. It is predicted that due to the increased aging in society, the incidence of AD will increase [1,2].

In the early stages of the disease, there are slight lapses in memory and problems with remembering new facts, but long-term memory is not affected. As the disease progresses, the cells responsible for memory and orientation undergo degeneration, there is increasing damage to the cerebral cortex, and, consequently, problems with speech, thought and memory loss. Long-term memory becomes damaged, and eventually brain atrophy occurs. The disease develops very slowly, and over time, the patient becomes unable to function without the help of a caregiver. Disorientation in time and space, mood changes, apathy, and an increased risk of depression frequently occur in patients. Progressive disorders lead to problems with recognizing family members. As dementia progresses, insomnia, irritability, delusions, and even aggressive behavior can occur. In advanced dementia, the patient is unable to carry out daily activities and has problems with physiological functions [3]. In developed countries, the disease is one of the most costly for society, covering many levels: social, psychological, physical and economic. There are currently no known treatments for advanced AD capable of entirely curing the disease. It is only possible to slow down its course and alleviate some of the symptoms. It is important to search for new diagnostic markers in order to effectively diagnose the disease at an early stage and to introduce appropriate treatment procedures.

Familial AD accounts for 5–10% of all cases, while sporadic AD accounts for 90–95% of cases. Although some GWAS studies have identified that some genes, such as ApoE4 and TREM2, significantly increase the risk of AD, the causes of sporadic AD are not fully understood. There are several hypotheses about the development of the disease. The oldest is the cholinergic hypothesis, which takes into account the decreased synthesis of the neurotransmitter—acetylcholine. Another is the amyloid hypothesis, according to which β-amyloid (Aβ) deposits play a fundamental role in the pathogenesis of the disease. According to an updated hypothesis, from 2009, Aβ plaques play a complementary role by impairing synaptic function. The amyloid hypothesis suggests that Aβ deposits play a pivotal role in the pathogenesis of the disease. Moreover, the specific apolipoprotein isoform, APOE4, is one of the major genetic risk factors for AD. Apolipoprotein increases the breakdown of Aβ, but some forms of apolipoprotein, such as APOE4, do this ineffectively. As a result, an excessive amount of amyloid builds up in the brain. In addition, rare variants of the myeloid-expressed trigger receptor 2 (TREM2) have been shown to increase the risk of developing AD. This receptor is a transmembrane receptor expressed in cells of the myeloid lineage. Another hypothesis is that abnormal phosphorylation of tau protein leads to NFT, which in turns lead to neuronal dysfunction and death. The difference between the amyloid and tau hypothesis is whether it is amyloid or tau that is the main cause or driver of the disease. According to another theory, AD may cause an age-related breakdown of myelin in the brain [1,2,3,4]. Moreover, misfolded and aggregated proteins bind to astroglial and micro-pattern recognition receptors, triggering an innate immune response. It is characterized by the release of inflammatory mediators that contribute to disease progression. Genome analysis suggests that several genes that increase the risk of sporadic AD encode for factors that regulate the clearance of misfolded proteins from glial cells and the inflammatory response. Comorbidities such as systemic inflammation and obesity can interfere with the immune processes in the brain, promoting disease progression [5].

In recent years, several other hypotheses have been put forward. Brain hypoperfusion associated with neurovascular dysfunction has been shown to be one of the causes of dementia in AD [6]. Moreover, it is believed that oxidative stress and reactive oxygen species may play an important role in the pathophysiology of strokes and cancer, but also in the development of dementia in AD [7,8]. One of the popular methods reflecting the concentration of enzymatic substances and other antioxidants is the Total Antioxidant Status (TAS). This method evaluates the combined antioxidant effects of all ingredients, including vitamins, lipids, proteins, uric acid, and glutathione [9].

Fibronectin is a protein dimer composed of two of the same monomers linked by a disulfide bridge. Each monomer has multiple sites binders, e.g., to collagen, heparin, integrin. It has a structural function, but also regulates the cell–matrix interaction. The basic molecular interaction of fibronectin with cells is the interaction with integral proteins of cell membranes, involved in the cell-matrix or cell-cell adhesion processes [10,11]. One of the functions of fibronectin is to transmit a signal from the external environment to the internal environment (inside the cell) [12]. The extracellular matrix (ECM) plays a key role as both a structural scaffold and regulator of cell signal transduction in tissues. In times of ECM assembly and turnover, cells upregulate the assembly of the ECM protein, fibronectin (FN). FN is assembled by cells into viscoelastic fibrils that can bind upward of 40 distinct growth factors and cytokines. These fibrils play a key role in assembling a provisional ECM during embryonic development and wound healing. Fibril assembly is also often upregulated during disease states, including cancer and fibrotic diseases. FN fibrils have unique mechanical properties, which allow them to alter mechanotransduction signals sensed and relayed by cells. The binding of soluble growth factors to FN fibrils alters the signal transduction from these proteins, while the binding of other ECM proteins, including collagens, elastins, and proteoglycans, to FN fibrils facilitates the maturation and tissue specificity of the ECM. 

Another function of fibronectin is in wound healing, clot formation and cellular immunity [13]. Research by Zlokovic et al. [14] shows that the dysfunction of the neurovascular system contributes to the development of AD. Defective clearance of Aβ peptide across the blood-brain barrier (BBB), aging of the cerebrovascular system, and abnormal angiogenesis may result in the dissociation of blood vessels, cerebral hypoperfusion, and neurovascular inflammation. Neuroinflammation may impair neurovascular function and increase BBB permeability [15]. In vitro studies by Martin et al. have shown that fibronectin can increase the amount of phosphorylated tau protein by up-regulation [10].

As the body ages, the amount of fibronectin matrix protein plays a special role in remodeling the microenvironment and abnormal neuronal activity [16]. In AD, fibronectin builds up in the senile plaques of the patient’s brain, most likely because it is partially the response of activated astrocytes to the presence of Aβ. Studies have shown that adding Aβ peptide to cultured astrocytes increases fibronectin [17].

Matrix metalloproteinases (MMPs) are enzymes that degrade extracellular matrix proteins during tissue modelling and repair [18], but also process bioactive molecules. It is likely that MMPs such as MMP-1 may affect collagen in the basal lamina of arterioles and cause vascular insufficiency seen in AD [19] as a result of breaking the blood-brain barrier which is common in AD.

The aim of the study was to evaluate the concentration of fibronectin and MMP-1 in patients with AD, as well as the influence of eating habits on the above parameters. Additionally, the relationship between the concentration of antioxidant elements in the serum and the above markers was assessed. The determination of fibronectin and MMP-1 was performed by an array Surface Plasmon Resonance imaging (SPRI) technique with specific biosensors. The SPRI is a label-free technique which was developed for the determination of protein biomarkers in body fluids without signal enhancement or preliminary preconcentration. Biosensors for the determination of MMP-1 [20] and fibronectin [21], with methods for biomarker determination, have been recently published. 

## 2. Materials and Methods

### 2.1. Characteristics of the Study Group

The study included 110 patients with early or moderate AD. The mean age was 77.8 ± 7.6 years (Table 1). The diagnosis was made according to criteria of the National Institute of Neurologic, Communicative Disorders and Stroke/Alzheimer’s Disease and Related Disorders Association (NINCDS-ADRDA), revised in 2007 [22]. 

**Figure 1:**
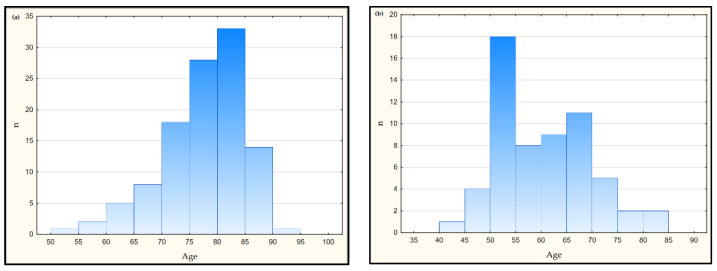
Scatterplot for age in the AD (**a**) and control groups (**b**)—statistically significant differences between groups: study group vs. control group (Mann-Whitney U test test), *p* < 0.001.

The control group consisted of 60 healthy people, with a mean age of 57.0 ± 7.9 years (Table 1). These people were not diagnosed with cognitive disorders and were professionally active. The exclusion criteria from participation in the study were: neoplastic diseases, autoimmune diseases, and diabetes (type 1 and 2). Figure 1 shows the age distribution in the study and control groups. The remaining parameters concerning the studied group were characterized in our previous publication [23].

All participants gave their written consent to participate in this study. The study was approved by the Local Ethics Committee (R-I-002/210/2018).

### 2.2. Food-Frequency Questionnaires (FFQ)

In order to assess eating habits, the participants of the study, either alone or with the help of their caregivers, completed the food frequency questionnaires (FFQ)—this questionnaire was developed by Committee of Human Nutrition Sciences of the Polish Academy of Sciences. The questionnaire included the above-mentioned 36 groups of food products. Frequent consumption of the products included consumption from 12 to 30 days per month. The exceptions were fish, where frequent consumption is consumption from 4 to 12 times a month. Products consumed less frequently were classified as occasional consumption [24]. We characterized individual groups of assessed products in the previous study [23].

### 2.3. Collection of Blood

About 6 mL of blood was collected with a vacutainer tube—containing a clot activator (Becton Dickinson, Toulouse, France), and centrifuged for 10 min at approximately 1000× *g*. The serum samples were stored at −20 °C.

### 2.4. Determination of Fibronectin and MMP-1 

The MMP-1 (Wuhan USCN, Hubei, Wuhan, China) and plasma fibronectin (Sigma, Steinheim, Germany) concentrations were assessed using an array Surface Plasmon Resonance Imaging (SPRI) biosensor. This technique was developed for the label-free specific determination of protein biomarkers, such as MMP-1 and fibronectin, in body fluids without biomarker preconcentration or signal enhancement. 

All of the necessary steps in the preparation and optimization of the biosensor and applied apparatus have been described in papers [20,21]. Gold chips were manufactured, as described in the cited above papers. The gold surface of the chip was covered with photopolymer and hydrophobic paint. Chips were rinsed with ethanol and water and dried under a stream of nitrogen. They were then immersed in 20 mM of cysteamine (SIGMA, Steinheim, Germany) ethanolic solutions for 2 h and after being rinsed with ethanol and water, were dried again under a stream of nitrogen.

The rabbit anti-human matrix metalloproteinase-1 antibody (RayBiotech, Inc., Peachtree Corners, GA, USA) and the rabbit anti-fibronectin antibody (Sigma, Steinheim, Germany) were immobilized on the thiol monolayer under the suitable conditions. Cysteamine was used as the thiol linker. The antibody solutions were activated in a PBS buffer with NHS (250 mM) and EDC (250 mM) (BIOMED, Lublin, Poland). Activation of the antibody was conducted by adding the mixture of NHS and EDC (1:1) in a carbonate buffer solution (pH 8.5) into the antibody solution and with vigorous stirring for 5 min at room temperature. Next, 3 μL of this solution was placed on the active places with the amine-modified surface and incubated at 37 °C for 1 h. After this, the biosensor was rinsed with water. Next, serum samples (MMP-1: 2 × diluted samples patients with AD, 5 × diluted samples control; Fibronectin: 10,000 × diluted samples patients with AD, 5000 × diluted samples control) were placed directly onto the prepared biosensor. The volume of the sample applied on each measuring field was 3 μL. The time of the interaction with the antibody was a maximum of 10 min. The biosensor was washed with water and HBS-ES buffer solution pH = 7.4 to remove unbound molecules from the surface. SPRI measurements for protein array were performed, as described in the previous papers, and the schematic diagram apparatus is given in the paper [20]. In order to control the level of nonspecific binding, some of the places on the biosensor covered with buffer were used. The SPRI signal was measured at a fixed SPR angle on the basis of the registered images. The first image was taken after the immobilization of the antibody. Then, the second image was taken after interaction with MMP-1/Fibronectin. Fibronectin and MMP-1 concentration readings (after dilution of samples) were made from previously prepared calibration curves. For fibronectin, the linear range of the calibration curve is 5–400 ng/mL [21], while for MMP-1 it is 0.05–20 ng/mL [20].

The SPRI signal was obtained by the subtraction of the signal after and before interaction with a biomolecule, for each spot separately. The contrast values obtained for all pixels across a particular sample’s single spot were integrated. Then, the SPRI signal was integrated over the spot area. NIH Image J version 1.32 software was used to evaluate the SPRI images in 2D form and to convert of numerical signal to a quantitative signal.

### 2.5. Determination of Cu, Se, Zn Content and Total Antioxidant Status (TAS)

The exact preparation of serum samples was described in the previous publication [17]. 

The determination of the content of the beneficial elements in the serum, such as Cu, Se and Zn, was carried out by means of electrothermal (Cu, Se) and flame (Zn) methods of atomic absorption spectrometry (AAS) with Zeeman background correction at 324.8 nm, 196 nm, and 213.9 nm, respectively (Z-2000 instrument, Hitachi, Tokyo, Japan), as detailed in our earlier publication [23].

The total antioxidant status (TAS) in the serum of the patients was determined by spectrophotometric method at a wavelength of 600 nm (Cintra 3030, GBC, Melbourne, Australia). Ready-made test kits from Randox Laboratories Ltd. (Crumlin, UK) were used to assess TAS [25]. We have characterized the individual analytical steps earlier in the paper [23].

### 2.6. Statistical Analysis

Statistical analyzes of the obtained data were performed with the Statistica v.13.3 software (TIBCO Software, Inc., Palo Alto, CA, USA). The differences between the groups were assessed with the Mann-Whitney U test. The strength of the correlation was assessed on the basis of the Spearman’s rank test. *p* values < 0.05 were considered statistically significant differences. In order to evaluate the influence of eating habits on the level of fibronectin and MMP-1 in the studied patients, stepwise multiple linear regression was performed.

## 3. Results

### 3.1. Concentration of Fibronectin and MMP-1

The mean levels of fibronectin reported in patients with AD were 687.99 ± 225.08 µg/mL, while in the group of healthy people the levels were 252.29 ± 60.77 µg/mL, the median was significantly higher in patients with AD (652.06 vs. 268.31 µg/mL).

The analysis of serum fibronectin concentration in patients with AD showed a significantly higher median concentration than in the control group (652.06 vs. 268.31 µg/mL). Moreover, it showed that patients with AD are characterized by a significantly lower median MMP-1 (4.62 vs. 18.09 ng/mL) (Table 2).

**Figure 2:**
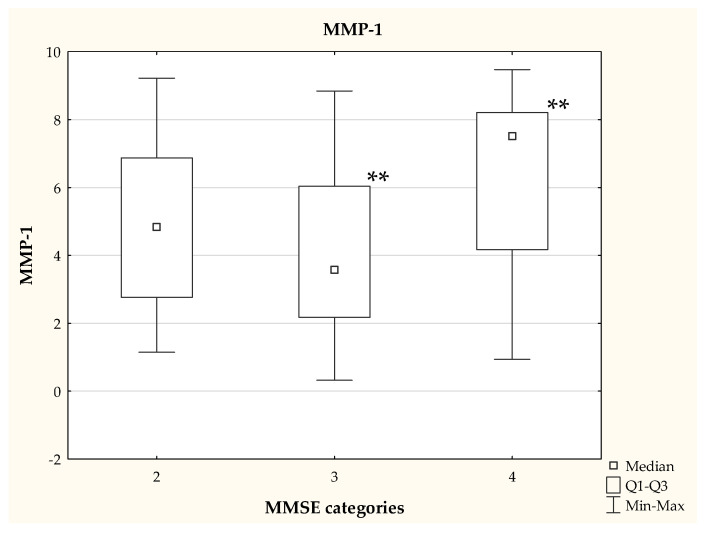
MMP-1 concentration depending on the MMSE category—data presented as medians, statistically significant differences between groups (Kruskal Wallis test), ** *p* < 0.01.

**Figure 3:**
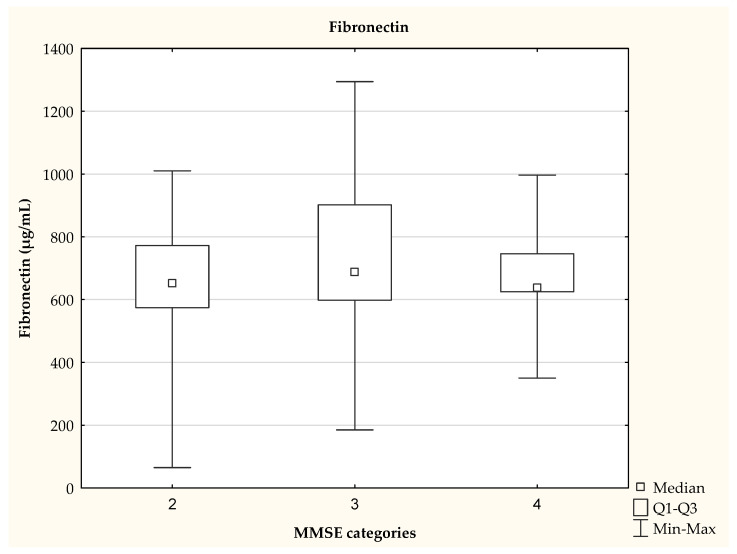
Fibronectin concentration depending on the MMSE category—data presented as medians, no statistically significant differences between groups (Kruskal Wallis test), *p* > 0.05.

We also observed that the patients classified into categories 3 and 4, on the basis of the MMSE test, differed in their MMP-1 concentration (*p* <0.01), as shown in Figure 2. The MMSE score was not significantly (*p* = 0.0997) associated with fibronectin levels (Figure 3).

### 3.2. Correlations between the Parameters Studied

In the case of patients with AD, we observed negative correlations between the concentrations of the tested elements with antioxidant properties and the concentration of MMP-1 (Table 3).

We found a positive correlation between the result obtained in the MMSE test and the concentration of MMP-1. Interestingly, a positive relationship was also found between the TAS and MMP-1 results (Table 3). There was no significant correlation between the concentration of the tested elements, TAS and the level of fibronectin.

### 3.3. The Influence of Eating Habit on Fibronectin and MMP-1 Concentration

A progressive multiple-step regression analysis was also performed. It was shown that 28% of serum fibronectin concentration may be dependent on the consumption of the products presented in Table 4, while the concentration of MMP may be dependent in 24%. The model explaining the effect of food products on the level of fibronectin included 13 groups of food products, while the models explaining the concentration of MMP-1 included 7 groups of food products (Table 4).

The regular consumption of fruit, flour dishes, meat, luxurious meats, fresh fish, and yellow and processed cheeses increases the concentration of fibronectin in serum. The consumption of white bread, wholemeal bread, sausages, poultry, potatoes, cold cuts and white cheese lowers the fibronectin concentration.

The consumption of wholemeal bread, white bread, sweet bread and white cheese reduces MMP-1 concentration. Increasing MMP-1 concentration can be caused by consumption of groats and rice, fruit and sausages.

## 4. Discussion

The patients included in our study had a significantly higher concentration of fibronectin compared to the healthy subjects. In 2009, Lemańska-Perek et al. [26] already indicated that the level of fibronectin may be an additional biomarker of AD. It has been shown that fibronectin in the plasma of elderly people both with and without dementia is present as a mixture of heterogeneous molecules with increasing molecular weights. The lower mass bands (about 240 kDa and about 220 kDa) have been shown to normally be present in plasma, while high molecular weight forms of FN (about 280 kDa and about 320 kDa) are observed in the plasma of AD subjects.

Studies by Lepelletier et al. (2017) [27] also indicated an increase in fibronectin levels in patients with AD. The authors divided the group of 30 patients into 3 groups (I: control group, II: patients with subclinical AD and III: patients with AD). In addition to fibronectin levels, ECM levels of collagen IV, perlecan, and human platelet endothelial cell adhesion molecule (hPECAM) were also assessed. The authors demonstrated increased expression of collagen IV, perlecan and fibronectin in patients with subclinical AD and AD, compared to the control group, in the frontal and temporal cortex, while no further increase was detected between subclinical AD and AD.

We have demonstrated a decrease in serum MMP-1 in patients with AD. Our studies showed a significant decrease of MMP-1 in the serum of AD patients. A study by Erhardt et al. (2021) [28] identified the most important biomarkers of mixed dementia in AD. Among them, three proteases have been identified: matrix-10 metalloproteinase—MMP-10, MMP-3 and MMP-1. Moreover, the role of three angiogenic factors (VEGF-C, Tie-2 and PLGF) and three cytokines was emphasized: interleukin-2 (IL-2), IL-6, IL-13.

Data on the role of MMP-1 are ambiguous. It has been suggested that the increased activity of MMP-1 in AD may contribute to dysfunction of the blood-brain barrier [29]. In contrast, Lorenzl et al. (2008) [30] emphasized that MMP1 does not correlate with the diagnosis of AD or with risk factors for future development of AD. The authors assessed the gelatinolytic activity (MMP-2 and MMP-9) in plasma samples using the zymographic method, while MMP-2, MMP-9, MMP-1, as well as TIMP-1 and TIMP-2, were measured by ELISA. The authors showed no significant changes in MMP-1 and MMP-2 levels; however, they indicated a significant increase in MMP-9 in the plasma of AD patients (*p* = 0.004) compared to controls.

An interesting issue is the negative correlations between antioxidant elements and MMP-1 in the group of patients. These patients are characterized by reduced serum concentrations of elements with antioxidant properties, which we have demonstrated in previous studies [23]. However, further research is needed to explain the reason for such a correlation.

The analysis of the relationship between eating habits and the level of fibronectin and MMP-1 indicates that patients should limit the consumption of fructose-rich (fruit), fatty and processed foods (cheese and processed cheese) and meat, as well as bread and flour products.

Excessive fructose intake is not recommended in AD. The phenomena of cerebral insulin resistance and mitochondrial dysfunction are emphasized, as well as the fact that the metabolism of brain fructose is a key pathway initiating AD. Moreover, inhibition of intracerebral fructose metabolism may be a new way to prevent and support therapy [31].

Johnson et al. [31] distinguished 5 steps linking fructose and the occurrence of AD: endogenous fructose is produced in the brain → fructose is metabolized, setting off an innate survival pathway → intracellular uric acid induces neuroinflammation → cerebral insulin resistance and glucose hypometabolism → formation of amyloid plaques and neurofibrillary tangles. 

Meat consumption is associated with an increased risk of dementia. This was confirmed in a large study of 493,888 people. The follow-up period was 8 ± 1.1 years. In this population, the participants were diagnosed with general dementia (*n* = 2896), AD (*n* = 1006) and vascular dementia VD (*n* = 490). The results of the study by Zhang et al. [32] showed that each additional consumption of 25 g/day of processed meat was associated with an increased risk of all forms of dementia. Increasing unprocessed meat consumption by 50 g/day was associated with a reduced risk of all forms of dementia and AD. A non-significant linear trend was shown for total meat and unprocessed poultry.

Increasingly, the relationship between the frequent consumption of highly processed foods and the deterioration of cognitive functions is emphasized. In 2022, the results of the study from the National Health and Nutrition Examination Survey (NHANES) 2011-14, including 3632 participants aged 69.1 ± 0.2 years, were published. The mean BMI was 29.0 ± 0.2 kg/m^2^. Cognition was assessed with a consortium in order to establish the Alzheimer’s Disease Registry (CERAD). In addition, a word learning test, an Animal Fluency test, and a Digit Symbol Substitution Test (DSST) were performed. Worryingly, highly processed foods accounted for an average of 53% of total energy consumption, ranging from 33 to 70%. Consumption of this type of food was significantly (*p* < 0.05) associated with lower results in the Animal Fluency test (the person is asked to name as many animals as possible within 60 s, for one animal they receive one point) in the elderly, without previous diseases [33].

It is emphasized that reducing the consumption of highly processed foods may be one way to improve cognitive impairment in the elderly.

White, wheat bread is included in this consumption, among other gluten. In 2021, the results of studies on the impact of gluten consumption by healthy women (*n* = 13,494) on their cognitive functions were published. Women participated in the Nurses’ Health Study II cohort study conducted in the USA. Nutrition data were obtained in the years 1991–2015, while cognitive functions were assessed in the years 2014–2019. Cognitive function was assessed by the validated assessment of the Cogstate Brief Battery: Psychomotor Speed and Attention Score, Learning and Working Memory Score, and Overall Cognitive Score. The mean gluten intake was 6.3 ± 1.6 g/day [34]. However, the authors showed no differences in cognitive functions between people with lower and higher intakes. The main sources of gluten were refined grains and whole grains. These results indicate that dietary gluten restriction is not required to maintain cognitive function in the absence of celiac disease or diagnosed gluten sensitivity.

Literature data indicate that changes in MMP-1 and fibronectin concentration may sometimes be non-specific. Unlike in AD, high levels of MMP-1 in plasma are associated with a poorer prognosis in colon cancer, as assessed by a multivariate analysis of 5-year survival [35]. Elevated serum levels of MMP-1 occurring in hypertension may be associated with increased collagen degradation in cardiovascular disease in the extracellular matrix [36]. In addition, it has been shown that cigarette smoke can affect the release of matrix metalloproteinase-1 (MMP-1) and tissue inhibitor of metalloproteinases type I (TIMP-1) in the lungs [37]. Logvinov et al. showed that the increase in blood pressure in rats on a high-fat and high-carbohydrate diet is caused, inter alia, by the release of fibronectin [38].

We found a positive correlation between fibronectin concentration and age in the control group (r = 0.42, *p* = 0.005). This may indicate that the concentration changes may be partially due to the aging of the organism, however, this requires further analysis.

There are also limitations to this study. When taking blood from healthy people, no data on BMI and eating habits were obtained. Moreover, healthy people did not have the MMSE test performed. We did not have data on eating habits in the group of healthy people. In future studies, it is necessary to match the test group to the control group in terms of age, as the age difference between the groups is the biggest limitation of our study. The age difference between the two study groups resulted in the difficulty of finding an appropriate number of healthy elderly people without other chronic diseases, which could affect the results obtained. Moreover, the sensitivity and specificity of the tested parameters in comparison to other disease entities should be determined.

## 5. Conclusions

Patients with Alzheimer’s disease have increased levels of fibronectin and decreased levels of MMP-1, which may be associated with impaired cognitive functions. Eating habits may, to some extent, affect the concentration of these markers, therefore an important element of prevention may be a properly balanced diet.

## Figures and Tables

**Table 1:** Characteristics of the study and control groups.

Parameter	Study Group Av. ± SD Min–Max (*n* = 110)	Control Group Av. ± SD Min–Max (*n* = 60)
Age	77.8 ± 7.6 54.0–93.0	57.0 ± 7.9 42.0–83.0
BMI	26.44 ± 4.13 17.78–40.23	nd
MMSE	20.4 ± 4.3 11–26	nd

Av.—average, nd—no data, SD—standard deviation.

**Table 2:** The concentration of fibronectin and MMP-1 in the study and control group.

Parameter (Units)	Study Group Av. ± SD Min–Max Med. (Q1–Q3)	Control Group Av. ± SD Min–Max Med. (Q1–Q3)	*p*-Value
Fibronectin (µg/mL)	687.99 ± 225.08 64.82–1294.13 652.06 (586.43–893.93)	252.29 ± 60.77 62.44–334.31 268.31 (199.71–295.19)	<0.00001
MMP-1 (ng/mL)	4.86 ± 2.68 0.32–9.47 4.62 (2.50–7.47)	17.49 ± 3.32 11.61–25.35 18.09 (15.47–19.09)	<0.00001

Av.—average, SD—standard deviation.

**Table 3:** Significant correlations between the studied parameters in the control group and the study group.

	Study Group	Control Group
Parameter 1 & Parameter 2	R (*p*-Value)	R (*p*-Value)
MMP-1 & Cu	−0.35 (0.0002)	ns
MMP-1 & Se	−0.40 (0.00002)	ns
MMP-1 & Zn	−0.22 (0.02)	ns
MMP-1 & MMSE	0.23 (0.03)	ns
MMP-1 & TAS	0.20 (0.04)	ns
Fibronectin & age	ns	0.42 (0.005)

ns—no significant, TAS—total antioxidant status.

**Table 4:** Stepwise linear regression analysis of multiple effects of food consumption frequency on the concentration of fibronectin and MMP-1 in the serum of patients with Alzheimer’s disease.

Independent Variables	β Coefficient (SE)	Significance Level	Adjusted R^2^
Fibronectin			
Fruit	0.369 (0.133)	0.008	0.28
Flour dishes	0.264 (0.126)	0.041	
White bread	−0.289 (0.137)	0.041	
Meat	0.241 (0.129)	ns	
Luxurious meats	0.239 (0.129)	ns	
Yellow and processed cheeses	0.188 (0.123)	ns	
Fresh fish	0.174 (0.122)	ns	
Wholemeal bread	−0.231 (0.146)	ns	
Sausages	−0.210 (0.123)	ns	
Poultry	−0.181 (0.133)	ns	
Potatoes	−0.178 (0.130)	ns	
Cold cuts	−0.174 (0.125)	ns	
White cheese	−0.135 (0.126)	ns	
MMP-1			
Groats, rice	0.350 (0.112)	0.002848	0.25
Wholemeal bread	−0.424 (0.131)	0.002031	
White bread	−0.308 (0.127)	0.018732	
Fruit	0.231 (0.117)	ns	
Sausages	0.200 (0.113)	ns	
Sweet bread	−0.220 (0.110)	ns	
White cheese	−0.202 (0.113)	ns	

ns—no significant.

## Data Availability

The data presented in this study are available on request from the corresponding author. The data are not publicly available due to privacy of ethical.
